# Association between rectus femoris cross-sectional area and diaphragmatic excursion with weaning of tracheostomized patients in the intensive care unit

**DOI:** 10.5935/0103-507X.20220087-en

**Published:** 2022

**Authors:** Fernando Nataniel Vieira, Raquel Bortoluzzi Bertazzo, Gabriela Carvalho Nascimento, Mariluce Anderle, Ana Cláudia Coelho, Fabiana de Oliveira Chaise, Jaqueline da Silva Fink, Wagner Luis Nedel, Bruna Ziegler

**Affiliations:** 1 Postgraduate Porgram in Pulmonological Sciences, Universidade Federal do Rio Grande do Sul - Porto Alegre (RS), Brazil.; 2 Intensive Care Unit, Grupo Hospitalar Conceição - Porto Alegre (RS), Brazil.; 3 Physiotherapy Unit, Hospital de Clínicas de Porto Alegre, Universidade Federal do Rio Grande do Sul - Porto Alegre (RS), Brazil.

**Keywords:** Tracheostomy, Ultrasonography, Ventilator weaning, Muscular atrophy, Diaphragm

## Abstract

**Objective:**

To verify the relationship between the rectus femoris cross-sectional area and diaphragmatic excursion with successful weaning from mechanical ventilation in chronic critically tracheostomized patients.

**Methods:**

This was a prospective observational cohort study. We included chronic critically ill patients (those who underwent tracheostomy placement after 10 days under mechanical ventilation). The rectus femoris cross-sectional area and diaphragmatic excursion were obtained by ultrasonography performed within the first 48 hours after tracheostomy. We measured rectus femoris cross-sectional area and diaphragmatic excursion to assess their association with weaning from mechanical ventilation, including their potential to predict successful weaning and survival throughout the intensive care unit stay.

**Results:**

Eighty-one patients were included. Forty-five patients (55%) were weaned from mechanical ventilation. The mortality rates were 42% and 61.7% in the intensive care unit and hospital, respectively. The fail group in relation to the success group at weaning presented a lower rectus femoris cross-sectional area (1.4 [0.8] *versus* 1.84 [0.76]cm2, p = 0.014) and lower diaphragmatic excursion (1.29 ± 0.62 *versus* 1.62 ± 0.51cm, p = 0.019). When rectus femoris cross-sectional area ≥ 1.80cm2 and diaphragmatic excursion ≥ 1.25cm was a combined condition, it had a strong association with successful weaning (adjusted OR = 20.81, 95%CI 2.38 - 182.28; p = 0.006) but not with intensive care unit survival (adjusted OR = 0.19, 95%CI 0.03 - 1.08; p = 0.061).

**Conclusion:**

Successful weaning from mechanical ventilation in chronic critically ill patients was associated with higher measurements of rectus femoris cross-sectional area and diaphragmatic excursion.

## INTRODUCTION

Mechanical ventilation (MV) is a life-support resource; however, there is an increase in mortality related to its duration, often due to complications such as ventilator-associated pneumonia and muscular dysfunction.^(1,2)^ Stopping ventilatory support is part of the routine of the intensive care unit (ICU), and it has been the focus of numerous studies; however, it remains surrounded by uncertainties,^(3,4)^ making it a complex issue.^(4)^

In critically ill patients who undergo prolonged MV, the placement of the tracheostomy (TCT) is a procedure often used to manage the weaning process. It is estimated that between 4 and 13% of mechanically ventilated patients require prolonged support, which is associated with an increase in health care costs, morbidity, and mortality.^(5)^ However, an ideal best practice recommendation for managing weaning from MV in chronic critically ill patients ventilated by TCT is still not well established.

Weaning parameters have been previously studied in mechanically ventilated patients recovering from acute respiratory failure and in tracheostomized patients. Several respiratory and peripheral muscle variables have been found to be associated with weaning from mechanical ventilation, and include cough strength,^(6)^ diaphragmatic strength,^(7)^ diaphragmatic thickness,^(8)^ diaphragmatic mobility,^(9)^ global respiratory muscle strength,^(10)^ handgrip strength,^(11)^ peripheral muscle strength,^(7)^ and peripheral muscle mass.^(12,13)^

Deficits in peripheral and respiratory muscle strength simultaneously occur in critically ill patients submitted to MV and are associated with its prolongation.^(14,15)^ Skeletal muscle mass is one of the factors that can reduce MV permanence in critically ill patients in the ICU,^(12)^ and diaphragmatic function has been considered as a marker for weaning from MV in these patients.^(9)^ Therefore, the bedside evaluation of the muscles in the ICU becomes important, and in this context, ultrasound is a useful, noninvasive, low-cost, and easily applicable tool,^(16)^ and more studies are required to validate its bedside applicability. Thus, the objective of this study was to verify the relationship between the rectus femoris cross-sectional area (RF-CSA) and the diaphragmatic excursion (DEx) with successful weaning from MV in chronic critically tracheostomized patients during their stay in the ICU.

## METHODS

A prospective cohort study was conducted in four adult ICUs in a public tertiary hospital in Brazil. The research was previously approved by the local ethics committee (protocol number 1942227). Informed consent forms were signed by the patients or their legal representatives.

The study sample consisted of patients admitted to the adult ICU, without a previous diagnosis of neuromuscular disease, who underwent TCT placement after 10 days under MV. Therefore, we characterized them as chronic critical patients, as proposed by Nelson et al.^(17)^

The RF-CSA and DEx were obtained by ultrasound performed within the first 48 hours after TCT. The Sonosite^®^ instrument (2013 SonoSite M-Turbo Model M-MSK) was used, and a trained intensivist performed the collection. Both measurements were performed by a researcher (FNV), and images were reviewed by another researcher (WLN).

Initially, the patient was placed in the supine position (30° elevation), relaxed, with the lower extremities extended and slightly apart. The image of the rectus femoris was obtained in the right lower extremity, frontally and perpendicularly, at the point representing one-third of the distance between the upper border of the patella and the anterior superior iliac spine.^(18,19)^ A two-dimensional ultrasound, B-mode and 10-5MHz (linear) transducer was used. After the muscle image was captured at the anatomical site, its cross-sectional area was expressed in square centimeters (cm^2^).

Diaphragmatic excursion was measured unilaterally (right dome of the diaphragm) during spontaneous ventilation with subjects in the same position as described above. The 5-2MHz (convex) transducer was positioned in the hepatic anatomical window between the middle clavicular line and the anterior axillary line, pointing medially, cranially, and dorsally, then projecting the ultrasound beam perpendicularly across the posterior third of the diaphragm. The DEx images were acquired with ultrasound in the M-mode. The vertical height measurement was recorded from the base of the inspiratory onset to the tipping apex at the end of the inspiration.^(20,21)^

Inspiratory muscle strength was measured through maximum inspiratory pressure (MIP), which was measured using the method proposed by Truwit et al.^(22)^ up to 48 hours after TCT placement. The digital manovacuometer (MVD 500, Globalmed®), which was connected to TCT followed by occlusion of the inspiratory branch for 20 - 25 seconds, was used. The highest value was recorded among the three reproducible measures.^(22,23)^

Medical records were obtained to collect the following data: age, sex, height, body weight, hospital length stay, ICU length of stay, duration of MV and duration of weaning from MV (in days), severity of the Simplified Acute Physiology Score III (SAPS III), Sequential Organ Failure Assessment Score (SOFA), reason for admission to the ICU and previous comorbidities.

The beginning of the weaning process had as a criterion the first spontaneous breathing test performed after the beginning of MV. The success in weaning of MV was defined within 48 free hours of MV for those who underwent MV for up to 20 days and 5 consecutive days without MV.^(24)^ for those who stayed in VM for a period of 21 days or more, which characterizes prolonged MV.^(5)^ Weaning failure was defined when the patient returned to MV (according to clinical decision) earlier than the periods described above or when weaning was suspended due to the palliative care being defined. Patients who were weaned from invasive MV for the use of bilevel positive airway pressure (BIPAP) MV through TCT were considered to have failed to wean.

The weaning from MV of tracheostomized patients was performed according to the decisions of the care team (respiratory therapist and attending physician), generally performing spontaneous breathing tests with progressive periods according to tolerance, intercalated with rest periods, until it was considered free from ventilatory support.

### Statistical analysis

The primary outcome of this study is the presence of successful weaning from MV. The data were presented as frequencies and proportions, means and standard deviations (SDs), or medians and interquartile ranges (IQRs). The normality of the variables was evaluated using the Shapiro-Wilk test. For comparisons between the groups according to successful weaning, Student’s t test was used for continuous variables with a normal distribution, and the Mann-Whitney *U* test was used for ordinal variables or data without a normal distribution. The association between continuous variables was made through the Pearson or Spearman correlation coefficient according to the normality of the distribution. The cutoff points for RF-CSA and DEx in relation to the success of weaning were defined through the receiver operating characteristic (ROC) curve and then the odds ratio (OR). Survival curves were plotted for the cumulative incidence of mortality from MV and ICU mortality according to muscular thresholds compared using the logarithmic rank test and the risk ratio. To assess the impact of each variable on the results, we performed a multivariate analysis using backward multinomial logistic regression. Collinearity between variables was analyzed in each regression performed. We included in each model distinct variables: RF-CSA, DEx, MIP, SOFA score on the first day of weaning, SAPS III on ICU admission, days in MV before TCT, and active infection at the beginning of the weaning process. The variables analyzed were selected for the model because they presented a p value < 0.20 in the univariate analysis compared to the different outcomes. Beyond RF-CSA and DEx, we also included distinct variables in each model due to their potential clinical relevance for weaning and perhaps confounders: MIP, SOFA score on the first day of weaning, SAPS III on ICU admission, days on MV before TCT, and active infection at the beginning of the weaning process. The variables with a p value < 0.20 remained in the model. In each model, we included RF-CSA and DEx as categorical variables, according to the cutoff points defined through the ROC curve. We performed a secondary analysis that included patients who achieved the RF-CSA and DEx cutoff points as a single categorical variable. We also performed an exploratory secondary analysis with two secondary outcomes: ICU death and palliative care establishment, with each variable isolated (RF-CSA and DEx) and with both variables as the same variable. The analysis was performed using the Statistical Package for Social Sciences (SPSS - IBM) version 21 and R (R Foundation) version 4.0.3. The level of statistical significance was established at p < 0.05.

The sample size was calculated according to the study by Dres et al.^(7)^ considering a significance level of 5% and a power of 80% to detect a difference of 0.5cm in the ExD variable. Considering a standard deviation of 0.42, 27 individuals are required in each group.

## RESULTS

From April 2017 to February 2018, 156 patients were placed on TCT throughout their stay in the ICU. Fifty-seven patients did not match the inclusion criteria, and 18 patients were excluded due to missing ultrasound measurements. After that, 81 patients were included in the study. Twenty-six patients (32%) had at least 21 days of MV at study inclusion; nevertheless, 65 subjects (80%) overcame this length of MV throughout their stay in the ICU.

Forty-five patients (55%) achieved successful weaning from MV at some point during their ICU stay. However, 36 patients (45%) remained dependent on some ventilatory support throughout the period. Among weaning failures, three patients were discharged from the ICU with BIPAP by TCT. Mortality in the ICU was 42% (34 cases), while hospital mortality was 61.7% (50 cases).

The general characteristics, comorbidities, and reasons for hospitalization in the ICU are presented in table 1. Palliative care was established in 28 cases (34.6%) of the sample. Although this action interrupted weaning from MV in 20 cases, 3 of them were subsequently weaned. Table 2 compares means or medians with characteristics or clinical situations according to success or failure in weaning.

**Table 1:** Sample general characteristics

Variables	
Male	46 (56.8)
Age (years)	67 (14)
SAPS III	76 ± 12
BMI (kg/m^2^)	26.3 (9)
Hospital length of stay (days)	55 (37.5)
ICU length of stay (days)	33 (17.5)
Total duration of MV (days)	30 (16.5)
Spent time on weaning from (days)	19 (16.5)
Comorbidities	
Pulmonary	36 (44.5)
Cardiac	22 (27)
Neurological	16 (20)
Renal	11 (13.6)
Oncological	8 (10)
HIV	2 (2.5)
Others	31 (38)
Reasons for ICU admission	
Sepsis (any source)	56 (69)
Pulmonary	50 (62)
Neurological	14 (17)
Cardiological	22 (27)
Abdominal	8 (10)
TCT motivation, other than prolonged MV	
Neurological impairment	32 (39.5)
Muscular weakness	31 (38)
Pulmonary function impairment	19 (23.5)
Persistent or active infection	12 (15)
Cardiac function impairment	10 (12)
Upper airway alterations	7 (9)

SAPS III - Simplified Acute Physiology Score III; BMI - body mass index; ICU - intensive care unit; MV - mechanical ventilation; HIV - human immunodeficiency virus; TCT - tracheostomy. The results are expressed as n (%), median (interquartile range) or mean ± standard deviation.

**Table 2:** Characteristics of tracheostomized patients according to success at weaning

Variables	Weaning successful		p value
	Yes (n = 45)	No (n = 36)	
Age (years)	68 (14.5)	66 (14.5)	0.527
BMI (kg/m^2^)	25.5 (8)	27.6 (9.6)	0.330
RF-CSA (cm^2^)	1.84 (0.76)	1.4 (0.8)	0.014
DEx (cm)	1.62 ± 0.51	1.29 ± 0.62	0.019
SAPS III	75.4 ± 13	76.8 ± 12	0.598
SOFA at TCT day	4 (3)	7.5 (5)	< 0.001
MIP (cmH2O)	-56 ± 28	-42 ± 16	0.004
Duration of MV before TCT (days)	16 (8.5)	19 (8)	0.067
Duration of MV after TCT (days)	6 (11.5)	16.5 (21)	< 0.001
Total duration of MV (days)	25 (12)	35 (23)	0.001
Time spent on weaning (days)	18 (17)	19 (16)	0.487
ICU length of stay (days)	33 (15.5)	33.5 (17.5)	0.118
Hospital length of stay (days)	59 (30.5)	45 (48)	0.079
ICU mortality	1 (2.2)	33 (91)	< 0.001
Hospital mortality	15 (33)	35 (97.2)	< 0.001

BMI - body mass index; RF-CSA - rectus femoris cross-sectional area; Dex - diaphragmatic excursion; SAPS III - Simplified Acute Physiology Score III; SOFA - Sequential Organ Failure Assessment; MIP - maximum inspiratory pressure; TCT - tracheostomy; MV - mechanical ventilation; ICU - intensive care unit. The results are expressed as the median (interquartile range), mean ± standard deviation or n (%).

The RF-CSA was significantly higher in patients who survived the ICU than in those who died (1.84 ± 0.81cm^2^ *versus* 1.39 ± 0.82cm^2^; p = 0.025). On the other hand, DEx did not present statistically significant differences (1.84 ± 0.55cm *versus* 1.36 ± 0.61cm; p = 0.143). Additionally, no significant difference was observed between sexes in the RF-CSA measurements (1.76 ± 0.89cm^2^ *versus* 1.66 [0.80] cm^2^; p = 0.226) or in the DEx measurements (1.51 [ 0.50] cm, *versus* 1.45 [0.66] cm; p = 0.657).

**Figure 1:**
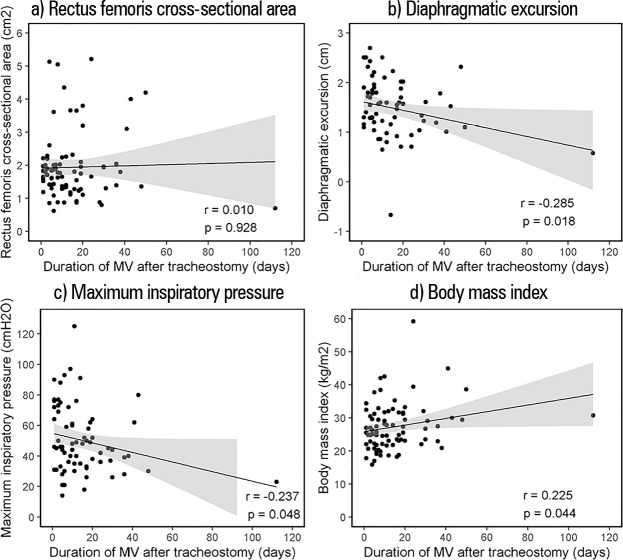
Correlations between the duration of mechanical ventilation after tracheostomy and (a) rectus femoris cross-sectional area, (b) diaphragmatic excursion, (c) maximum inspiratory pressure, and (d) body mass index.

There was a statistically significant positive correlation between RF-CSA and body weight (r = 0.416; p = 0.001), body mass index - BMI (r = 0.279; p = 0.012) and height (r = 0.333; p = 0.002) and a statistically significant negative correlation between DEx and SOFA score (r = -0.258; p = 0.033) and total duration of MV (r = -0.297; p = 0.014). Rectus femoris cross-sectional area and DEx did not show correlations with the following variables: age, duration of MV before TCT, length of stay in the ICU, hospital length of stay, SAPS-3, and MIP (p > 0.05). Furthermore, there was no correlation between RF-CSA and DEx (r = 0.037; p = 0.763). The correlations between the duration of MV after TCT placement and RF-CSA, DEx, MIP and BMI are illustrated in figure 1.

MV - mechanical ventilation.

**Figure 2:**
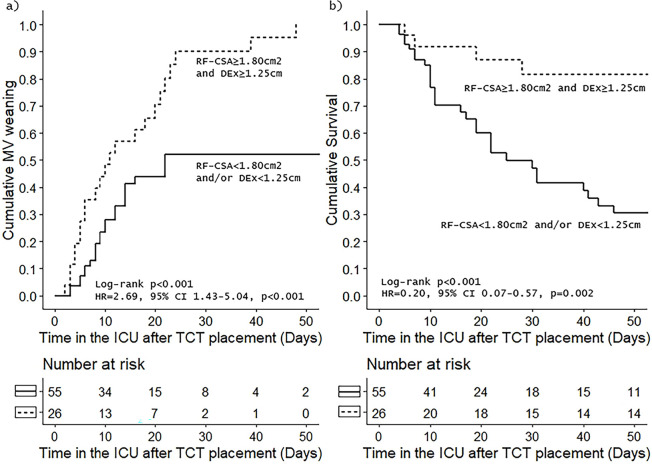
Survival plots according to rectus femoris cross-sectional area and diaphragmatic excursion of time from tracheostomy placement: (a) cumulative incidence of weaning from mechanical ventilation and (b) cumulative survival in the intensive care unit.

Successful weaning from MV was associated with RF-CSA ≥ 1.80cm^2^ (OR = 3.41; 95%CI 1.35-8.61; p = 0.008) and DEx ≥ 1.25cm (OR = 3.31; 95%CI 1.20 - 9.15; p = 0.019). However, the highest association with MV release was observed when both muscular thresholds were hit by the same patient (OR = 11.5; 95%CI 3.08 - 42.99; p < 0.001). Furthermore, these conditions also increased the odds of survival in the ICU (OR = 6.6; 95%CI 2.01 - 21.70; p < 0.001). Figure 2 shows survival plots according to the presence or absence of RF-CSA and DEx thresholds reached simultaneously according to the risk of failure at MV weaning attempts in the ICU and to the risk of death in the ICU.

MV - mechanical ventilation; RF-CSA - rectus femoris cross-sectional area; DEx - diaphragmatic excursion; HR - hazard ratio; 95% CI - 95% confidence interval; ICU - intensive care unit; TCT - tracheostomy.

In multivariate analysis, RF-CSA ≥ 1.80cm^2^ but not DEx ≥ 1.25cm was an independent variable associated with weaning success (adjusted OR [aOR] = 5.85; 95%CI 1.22 - 28), in addition to MIP and SOFA score at the first day of weaning. In another model exploring the same outcome, when RF-CSA ≥ 1.80cm^2^ and DEx ≥ 1.25cm were combined in a single categorical variable, it was associated with weaning success (aOR 20.81 (2.38 - 182.28), in addition to MIP and SOFA score at the first day of weaning. Neither RF-CSA ≥ 1.80cm^2^ nor DEx ≥ 1.25cm, individually or in combination, was associated with ICU mortality, and only MIP (in both models) was associated with the outcome. Diaphragmatic excursion < 1.25cm was an independent predictor of palliative care and was further established, in addition to the SOFA score on the first day of weaning and SAPS III at ICU admission. Diaphragmatic excursion ≥ 1.25cm in combination with RF-CSA ≥ 1.80cm^2^ was also associated with palliative care being established. In this modeling, the SOFA score on the first day of weaning and SAPS 3 at ICU admission were also associated with the outcome.

## DISCUSSION

In our study, we found an association between RF-CSA and DEx with the success of weaning from MV in a population of critical illnesses submitted to TCT. Ultrasound-detected diaphragm dysfunction has previously been studied, with controversial results in terms of association with weaning from MV,^(9,25,26)^ due to a wide range of precision variation between populations and studies. Moreover, there remains a lack of evidence on this topic in chronic critically ill patients submitted to TCT. In this study, a DEx ≥ 1.25cm was not associated with successful weaning in this population when adjusted for confounding factors. Rectus femoris cross-sectional area ultrasound evaluation is considered to be a simple, noninvasive, and easily reproducible bedside method and can be used as a marker of peripheral muscle wasting in critically ill patients during the ICU stay. ^(27-31)^ Muscle wasting in this context is associated with worse patient-centered outcomes,^(28-30)^ and RF-CSA may indirectly infer muscle reserve.^(31)^ In our data, a cutoff measurement of RF-CSA ≥ 1.80cm^2^ was an independent predictor of weaning success but was not associated with lower ICU mortality. Respiratory muscles and skeletal muscles are strongly affected by critical illnesses that contribute to prolongation of MV and failure of weaning.^(7,14,15,32)^ When these variables were combined, the prediction power for successful weaning was improved in our study.

High mortality rates and weaning failures are widely studied outcomes in critically ill patients.^(33-39)^ In this study, the failure rate for weaning from MV was 45%. The group of patients with weaning failure had a mortality rate of 91.6% in the ICU and 97.2% in the hospital. These failure rates for weaning,^(35)^ as well as the ICU and mortality rates, are similar to the rates in previous data.^(36)^ This population is the hallmark of chronic critical illness,^(40)^ and muscle dysfunction is one of the most easily noticeable dysfunctions in these patients. Discussion about palliative care, with family members, caregivers, and patients themselves, is inseparable from the weaning process of whole care. A major limitation of our study is that we were unable to differentiate between patients who progressed to palliative care implementation because they were unable to progress with the process of weaning from MV or due to other causes. In this context, we chose to analyze the decision to implement exclusive palliative care as an outcome to demonstrate its association with muscle variables. Since weaning failure is directly associated with high mortality in this population, it seems clear to us that RF-CSA ≥ 1.80cm^2^ and DEx ≥ 1.25cm are associated with a lower chance that the patient will progress to palliative care.

The main tool for the data collection of this study was ultrasonography for musculoskeletal evaluation. This has been gaining space in the ICU, as serious muscle damage resulting from critical illness has been shown to have a negative impact on outcomes.^(16,27)^ It is a noninvasive tool that does not expose the patient to radiation and is still easy to apply and reproducible at the bedside.^(16,18,41)^ Increasingly, it has shown a good capacity to provide quantitative and qualitative data on muscle conditions.^(9,19,26,27,41)^ It has been used in research and clinical practice, thus contributing to understanding the mechanisms of harmful muscle fibers and consequently to the development and implementation of strategies to prevent or recover muscular damage.^(42)^ In the future, these data should be prospectively analyzed. Therefore, DEx and RF-CSA measurements can be included in future clinical trials in the area. Different therapeutic strategies for weaning, motor activity, and eventually nutritional support according to initial measures are a promising field of research in this population. The progression of measurements at fixed time intervals should also become a future topic of investigation.

However, this study has some limitations. Primarily, it was carried out in a single center, so it was not possible to generalize our results. This study had a high number of patient exclusions, which also limited its generalizability to other populations. Furthermore, the absence of weaning and mobilization protocols may have led the patients to different management, since the medical decision is also subject to clinical judgment. Transversal evaluation performed at the beginning of the study does not allow inferences over time. Interobserver variability in measurements was minimized in this study, but it may be a concern in real-life scenarios. The lack of data on sedation and analgesia levels is another important limitation of this study. Prolonged weaning is commonly due to several variables, and we were unable to define isolated causes of prolonged weaning in our population. We did not quantify how many patients were placed in palliative care solely because they were unweanable from MV. This is a relevant limitation in this work, although it was minimized when analyzing this event as a secondary outcome. In addition, there are no normal prediction equations for RF-CSA, and this variable is known to be influenced by body weight, height, BMI, sex, and age.^(43)^ There are attempts to normalize these data with critical patients, multiplying the RF-CSA of women by the coefficient 1.484 to obtain the correction in relation to the male sex^(30)^ and comparing with healthy individuals.^(15)^

## CONCLUSION

The rectus femoris cross-sectional area and diaphragmatic excursion of chronic critically ill patients on mechanical ventilation who underwent tracheostomy were higher in the group that had successful weaning in the intensive care unit. Furthermore, the association between rectus femoris cross-sectional area and diaphragmatic excursion above 1.80cm^2^ and 1.25cm, respectively, is associated with greater success in weaning from mechanical ventilation, even when corrected for other potentially confounding variables.

## Figures and Tables

**Table 3:** Multivariate analysis exploring clinical and ultrasonographic variables and outcomes

	aOR (95%CI)	p value
Outcome - weaning success		
RF-CSA ≥ 1.80cm^2^ (reference: yes)^*^	5.85 (1.22 - 28)	0.027
DEx ≥ 1.25cm (reference: yes)^*^	2.11 (0.52 - 8.54)	0.295
MIP†	1.06 (1.01 - 1.11)	0.015
SOFA†	0.67 (0.5-0.9)	0.008
SAPS III†	0.96 (0.91 - 1.02)	0.162
Active infection ^*^ (reference: yes)	0.07 (0 - 1.14)	0.062
Outcome - weaning success		
RF-CSA ≥ 1.80cm^2^ plus DEx ≥ 1.25cm (reference: yes)^*^	20.81 (2.38 - 182.28)	0.006
MIP†	1.07 (1.01 - 1.12)	0.011
SOFA†	0.63 (0.46 - 0.86)	0.003
SAPS III†	0.95 (0.9 - 1.02)	0.139
Days on MV pre weaning (**≤** 21 days)^*^	3.66 (0.62 - 21.49)	0.151
Active infection^*^	0.08 (0 - 1.51)	0.091
Outcome - ICU mortality		
RF-CSA ≥ 1.80cm^2^ (reference: yes)^*^	0.32 (0.08 - 1.26)	0.096
DEx ≥ 1.25cm (reference: yes)^*^	1.35 (0.31 - 5.76)	0.687
MIP†	0.95 (0.9 - 0.99)	0.005
SOFA†	1.78 (1.3 - 2.44)	<0.001
SAPS III†	1.04 (0.98 - 1.1)	0.142
Outcome - ICU mortality		
RF-CSA ≥ 1.80cm^2^ plus DEx ≥ 1.25cm (reference: yes)^*^	0.19 (0.03 - 1.08)	0.061
MIP†	0.94 (0.9 - 0.99)	0.014
SOFA†	1.05 (0.99 - 1.11)	0.104
Days on MV pre weaning (< 21 days)^*^	0.28 (0.05 - 1.51)	0.139
Outcome - palliative care		
RF-CSA ≥ 1.80cm^2^ (reference: yes)^*^	0.59 (0.16 - 2.14)	0.427
DEx ≥ 1.25cm (reference: yes)^*^	0.18 (0.04 - 0.76)	0.019
MIP†	0.98 (0.95 - 1.01)	0.185
SOFA†	1.38 (1.08 - 1.76)	0.01
SAPS III†	1.07 (1.01 - 1.13)	0.019
Outcome - palliative care		
RF-CSA ≥ 1.80cm^2^ plus DEx ≥ 1.25cm (reference: yes)^*^	0.2 (0.05 - 0.84)	0.027
SOFA†	1.26 (1.05 - 1.51)	0.015
SAPS III†	1.06 (1.01 - 1.11)	0.013

aOR - adjusted odds ratio; 95%CI - 95% confidence interval; RF-CSA - rectus femoris cross-sectional area; DEx - diaphragmatic excursion; MIP - maximal inspiratory pressure; SOFA - Sequential Organ Failure Assessment; SAPS III - Simplified Acute Physiology Score III; ICU - intensive care unit; MV - mechanical ventilation.

* Categorical variables in the model; † continuous variables in the model.
